# Distal Ureteral Stricture Secondary to Urolithiasis: Stepwise Endourological and Surgical Management with Ureteral Reimplantation and Drug-Coated Balloon Dilation (Optilume)

**DOI:** 10.3390/life16040677

**Published:** 2026-04-15

**Authors:** Patricia Rodriguez-Parras, Ana Morales-Martinez, Alberto Zambudio-Munuera, Miguel Arrabal-Martin, Miguel Angel Arrabal-Polo

**Affiliations:** 1 Department of Urology, San Cecilio University Hospital, 18016 Granada, Spain; 2 Group Uromet, IBS Institute of Investigation, 18016 Granada, Spain

**Keywords:** ureteral stricture, urolithiasis, ureteral reimplantation, paclitaxel-coated balloon

## Abstract

Introduction: Acquired ureteral stricture is an uncommon but clinically relevant complication, mainly associated with long-standing urolithiasis, chronic inflammatory processes, and repeated endourological procedures. Case presentation: We present the case of a 48-year-old woman with left distal ureteral stricture secondary to urolithiasis and repeated endourological procedures, with a complicated clinical course and progressive renal functional impairment. Despite stepwise management including balloon dilations, endoscopic incision, prolonged urinary diversion, and ultimately ureteral reimplantation with a psoas hitch, the patient developed restenosis of the ureteral neomeatus. Due to persistent obstruction, endoscopic dilation with a paclitaxel-coated balloon (Optilume^®^) was performed. Subsequent imaging demonstrated partial improvement in ureteral drainage and relative functional improvement of the left kidney. Conclusion: This case highlights the potential complementary role of drug-coated balloons in complex and refractory benign ureteral strictures, although the currently available evidence remains limited.

## 1. Introduction

Acquired ureteral stricture is an uncommon but clinically relevant complication, mainly associated with long-standing urinary stone disease, chronic inflammatory processes, and repeated endourological procedures. Its management remains a therapeutic challenge, particularly in refractory cases, where progressive renal damage and the need for multiple interventions may coexist.

Initial treatment is usually conservative or endourological, including balloon dilation, endoscopic incision, and temporary urinary diversion with a double-J stent or percutaneous nephrostomy. However, restenosis rates after these procedures are high, particularly in long, distal strictures or those secondary to chronic inflammation, which often necessitates definitive reconstructive surgery, such as ureteral reimplantation [1].

In recent years, paclitaxel-coated drug-eluting balloons have emerged as a promising alternative for the treatment of benign ureteral strictures. Paclitaxel exerts a local antiproliferative effect that may reduce fibroblastic hyperplasia and, consequently, the rate of restenosis, a mechanism that has been widely validated in the vascular field. Although its use in urology remains limited, early clinical studies suggest favorable results in selected ureteral strictures [2].

In 2022, Kallidonis et al. published the first series with long-term follow-up on the use of paclitaxel-coated balloons in benign ureteral strictures, showing acceptable ureteral patency rates and a favorable safety profile [2]. Subsequently, isolated case reports have expanded its application to complex settings, such as ureteroileal anastomotic strictures, with encouraging preliminary results [3].

At present, the ENDURE1 study represents the only ongoing prospective clinical trial systematically evaluating the efficacy of the Optilume^®^ balloon in benign ureteral strictures, highlighting that the currently available evidence remains limited and is based mainly on small series and case reports [4]. A recent review from the European endourological field emphasized the potential of this technology, while underscoring the need for further data before widespread adoption [5].

We present the case of a patient with refractory distal ureteral stricture secondary to urolithiasis, treated with a stepwise approach that included multiple endourological techniques, reconstructive surgery and, finally, drug-coated balloon dilation, illustrating the potential role of this technology as a complementary option in complex scenarios.

## 2. Case Presentation

A 48-year-old woman, with no known drug allergies and a history of recurrent bilateral nephrolithiasis for several years, had previously undergone multiple urological procedures:

• Bilateral extracorporeal shock wave lithotripsy.
• Bilateral ureteroscopy in 2017 with stone extraction.
• Retrograde intrarenal surgery (RIRS) for a right lower calyceal stone in February 2018.

**Figure 1:**
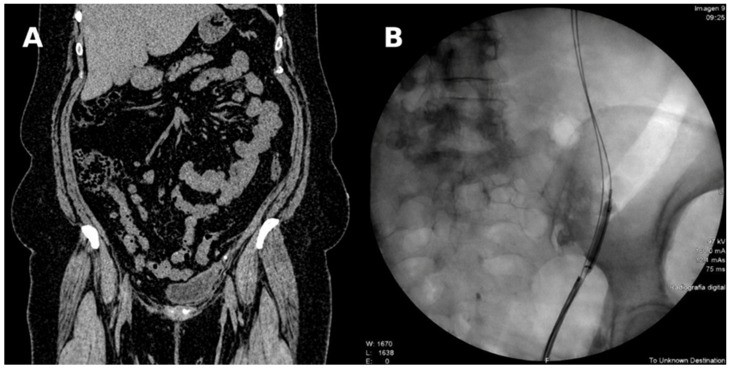
Initial evaluation and endourological treatment of the left distal ureteral stricture. (**A**) Non-contrast coronal abdominopelvic CT scan showing stones in the left pelvic ureter associated with grade IV ureterohydronephrosis and severe dilatation of the collecting system. (**B**) Intraoperative fluoroscopic image during ureteroscopy with pneumatic dilation and endoscopic incision of the distal ureteral stricture, with placement of a double-J stent.

In March 2023, she presented with left renal colic. A non-contrast abdominopelvic CT scan revealed two stones measuring approximately 3 mm in the left pelvic ureter, associated with grade IV ureterohydronephrosis (Figure 1A). A left double-J stent was placed at another outside institution.

The CT scan also showed renal asymmetry, with a right kidney measuring 12.2 cm and displaying pyelonephritic scars, and a left kidney measuring 15.8 cm with severe dilatation of the collecting system.

In November 2023, she presented to our institution, where left ureteroscopy was performed, revealing a distal ureteral stricture confirmed by retrograde pyelography. Pneumatic balloon dilation, endoscopic incision of the superior ureteral wall, and placement of a double-J stent were performed (Figure 1B).

After stent removal in February 2024, grade IV left ureterohydronephrosis persisted, with cortical atrophy and delayed contrast excretion. Isotopic renography showed a partially obstructive pattern in the left kidney, with a relative function of 41.7%.

In view of these findings, a left percutaneous nephrostomy and a new double-J stent were placed. In May 2024, combined pyelography demonstrated a 1 cm ureteral stricture at the sacral level, and repeat balloon dilation was performed.

After multiple episodes of restenosis documented on combined pyelography, a 2.5 cm filiform distal stricture was identified in October 2024, and definitive surgical treatment was decided upon. Given the complexity and prolonged clinical course, a detailed chronological summary of the patient’s management is provided in Table 1.

On 14 March 2025, a psoas hitch with left ureteral reimplantation was performed, leaving a double-J stent and percutaneous nephrostomy in place. After stent removal, distal obstruction persisted, requiring continued urinary diversion.

Subsequently, in November 2025, under sterile conditions, antegrade access was obtained through the existing nephroureteral catheter. Guidewire advancement was initially difficult due to catheter calcification; therefore, the distal end of the catheter was trimmed, allowing successful coaxial guidewire placement. A retrograde ureteroscopic approach was attempted but was not feasible due to lateral displacement and stenosis of the ureteral meatus. Consequently, an antegrade approach was adopted, and an access sheath was advanced from the nephrostomy tract across the neomeatus into the bladder.

Following successful access, a guidewire was advanced across the ureteral stricture into the bladder under fluoroscopic guidance. The paclitaxel-coated Optilume^®^ balloon (30 Fr, 5 cm length) was then advanced over the guidewire and positioned across the stenotic segment, ensuring that at least 5 mm of the balloon extended beyond both proximal and distal margins of the stricture to cover healthy ureteral tissue. Correct positioning was confirmed fluoroscopically using the radiopaque markers. The device was prepared with a 50:50 mixture of iodinated contrast and saline to allow visualization during inflation. Prior to full inflation, the balloon was left in position for approximately one minute to allow adequate surface hydration and optimize paclitaxel delivery.

The balloon was then gradually inflated using a pressure-controlled device to approximately 10 atm, according to manufacturer recommendations for a 30 Fr balloon. Inflation was maintained for 5 min to ensure adequate mechanical dilation and drug transfer to the ureteral wall. After completion of dilation, the balloon was fully deflated and carefully removed under fluoroscopic guidance.

**Figure 2:**
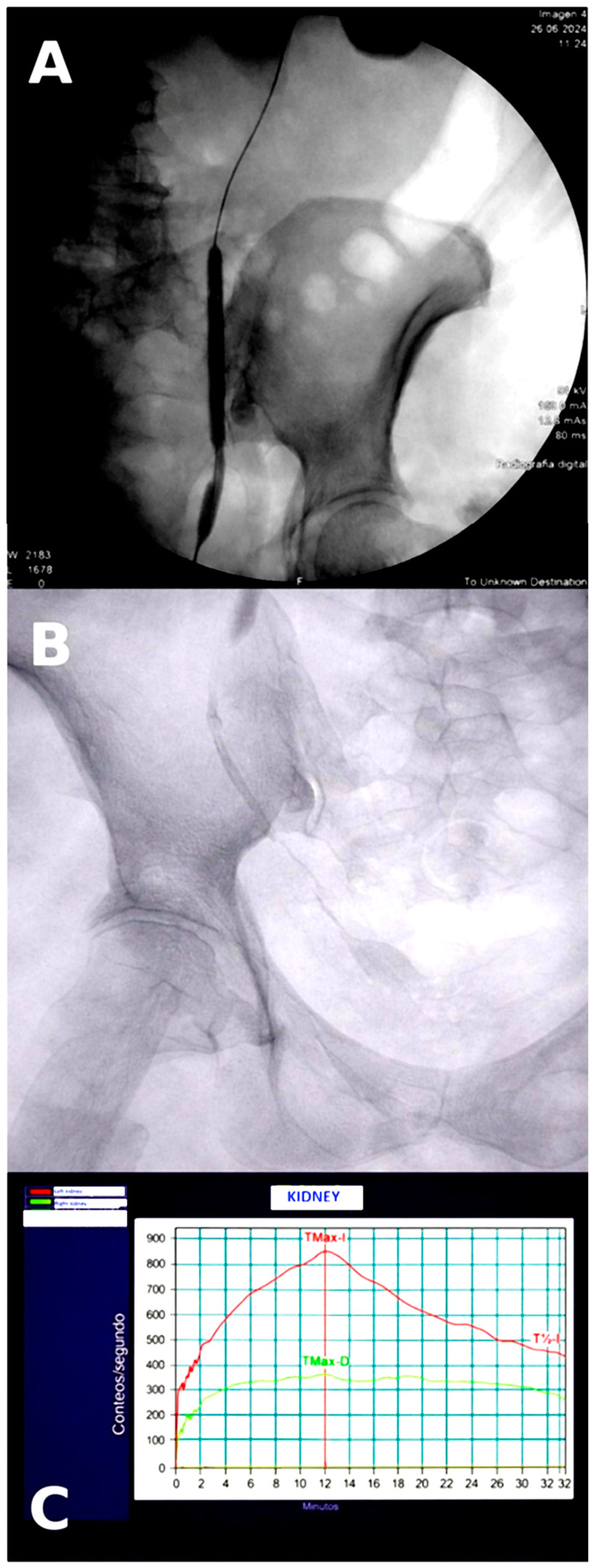
Management of postoperative restenosis and renal functional evolution. (**A**) Fluoroscopic image during endoscopic dilation with a paclitaxel-coated balloon (Optilume^®^) at the neo-ureteral meatus after ureteral reimplantation. (**B**) Nephrostogram with 50% diluted iodinated contrast showing passage of contrast into the bladder through a distal ureteral tract of reduced caliber, consistent with residual functional stricture. (**C**) Follow-up diuretic renogram (MAG-3) showing functional improvement of the left kidney, with increased relative renal function (66.2%) and a steeper excretory curve compared with the previous study.

A 6 Fr × 24 cm double-J stent was placed at the end of the procedure. A safety nephrostomy tube was left in place and maintained closed. Perioperative antibiotic prophylaxis was administered according to institutional protocol. The postoperative course was uneventful, with no intraoperative or early postoperative complications (Figure 2A).

Subsequent radiological follow-up showed partial improvement in ureteral passage, with no stricture at the ureteropelvic junction and persistent narrowing at the ureteral reimplantation site, with filiform passage into the bladder.

On nephrostography performed on 7 January 2026, contrast passage into the bladder was demonstrated, although through a narrower lumen, consistent with residual functional stricture at the reimplantation site.

The double-J stent placed after Optilume^®^ dilation was removed on 18 December 2025, at the time of a descending nephrostogram. All subsequent imaging studies, including nephrostograms performed in January and February 2026 and the diuretic renogram (MAG-3), were carried out without an internal ureteral stent, with the nephrostomy tube as the only drainage system in place.

A repeat nephrostogram was performed on 2 February 2026. Diluted iodinated contrast (50%) was administered through the nephrostomy, followed by fluoroscopy and radiographs, demonstrating passage of contrast into the bladder. The distal ureteral tract remained reduced in caliber, but contrast passage into the bladder was maintained (Figure 2B). The nephrostomy was left closed.

Finally, diuretic renography showed the following results: compared with the previous MAG-3 study (27 March 2024), right kidney function had significantly decreased, and the functional curve showed an obstructive pattern. The left kidney exhibited compensatory functional changes, with a steeper slope during the excretory phase. Relative renal function was 66.2% for the left kidney and 33.8% for the right kidney (Figure 2C).

This finding should be interpreted with caution, as relative renal function is a proportional parameter. The apparent decrease in right kidney function may reflect a redistribution phenomenon associated with improved drainage of the left kidney rather than true contralateral deterioration. No clinical or imaging findings suggested acute pathology affecting the right kidney.

At the most recent follow-up (7 April 2026), the patient remains asymptomatic and has not required any emergency department visits.

## 3. Discussion

This case illustrates the complexity of managing distal ureteral strictures in patients with repeated endourological interventions and progressive fibrosis. Despite a stepwise approach including balloon dilation, endoureterotomy, prolonged urinary diversion, and ultimately ureteral reimplantation, the patient developed recurrent stenosis at the neomeatus. This represents a challenging clinical scenario in which standard reconstructive strategies may fail and further surgical options may be associated with increased morbidity.

Benign ureteral strictures are most commonly related to inflammatory processes, urolithiasis, and prior instrumentation [6,7]. Although minimally invasive endourological techniques are usually the first-line approach, recurrence rates remain significant, particularly in long, distal, or highly fibrotic strictures [8]. In such cases, reconstructive surgery, including ureteral reimplantation, is considered the standard definitive treatment, with high success rates reported in the literature [9,10].

However, anastomotic restenosis after ureteral reimplantation, although uncommon, represents a particularly challenging situation. Therapeutic options in this setting are limited and may involve repeat endourological interventions or complex reoperative surgery, both of which may be associated with increased morbidity and uncertain outcomes [11].

In this context, the use of a drug-coated balloon was considered as a minimally invasive alternative before proceeding to more aggressive surgical options. The rationale for using Optilume^®^ lies in its combined mechanical dilation and local antiproliferative effect, which may reduce fibroblast activity and the risk of restenosis in highly fibrotic segments, particularly after reconstructive surgery [12].

Although clinical experience with paclitaxel-coated balloons in ureteral strictures remains limited, early reports suggest that this technology may improve ureteral patency in selected cases [2,3]. In the present case, post-procedural imaging demonstrated maintained, albeit reduced-caliber, ureteral passage, together with relative functional improvement of the affected kidney. However, these findings should be interpreted with caution, as changes in relative renal function may partially reflect contralateral functional deterioration rather than true absolute recovery [13].

This report has several limitations that should be acknowledged. First, it describes a single-patient experience, which limits generalizability. Second, no validated symptom scores or quality-of-life questionnaires were used, which restricts objective assessment of clinical benefit from the patient’s perspective. Third, follow-up after drug-coated balloon (DCB) dilation is relatively short and insufficient to assess long-term durability of ureteral patency.

Additionally, the response to treatment was partial, as imaging demonstrated persistent reduced-caliber ureteral passage despite maintained drainage. Furthermore, no urodynamic or pressure-flow studies were performed to objectively quantify the degree of obstruction or functional improvement. These factors should be considered when interpreting the clinical and functional outcomes of this case.

Further prospective studies are needed to better define the role of drug-coated balloons in the management of complex benign ureteral strictures [4].

## Figures and Tables

**Table 1:** Chronological timeline of clinical course, interventions, and outcomes (2023–2026).

Date	Clinical Event/Intervention	Stent/Nephrostomy Management	Imaging Findings	Renal Function
Mar 2023	Left renal colic → DJ stent placement	DJ stent (left)	CT: Grade IV hydronephrosis, distal ureteral stones	—
Nov 2023	URS + balloon dilation + endoureterotomy	DJ stent (left)	Stricture confirmed	—
Feb 2024	DJ removal	No drainage	—	—
Apr 2024	Follow-up evaluation	DJ + nephrostomy (left)	CT: persistent hydronephrosis; renogram: obstruction	Left 41.7%/Right 58.3%
May 2024	Balloon dilation	DJ stent (left)	Stricture ~1 cm	—
Jul 2024	DJ removal	No drainage	—	—
Sep 2024	Nephrostomy opened	Nephrostomy (left)	Filiform passage	—
Oct 2024	Combined pyelography	Nephrostomy	Distal stricture 2.5 cm	—
Mar 2025	Reimplantation + psoas hitch	DJ + nephrostomy	—	—
Apr 2025	DJ removal	Nephrostomy	Persistent obstruction	—
Apr 2025	Nephroureteral catheter	Nephroureteral catheter	—	—
Jul 2025	DJ removal	Nephrostomy reopened	—	—
Jul 2025	Descending pyelography	Nephrostomy	No contrast passage	—
Aug 2025	Right URS + laser	DJ (right) + nephrostomy (left)	Bilateral lithiasis	—
Aug 2025	Right DJ removal	Nephrostomy	—	—
Nov 2025	Optilume dilation	DJ + nephrostomy	—	—
Dec 2025	Follow-up CT	Nephroureterostomy	Grade III hydronephrosis	—
Dec 2025	DJ removal + nephrostogram	Nephrostomy	No passage	—
Jan 2026	Nephrostogram	Nephrostomy	Partial passage	—
May 2026	URS + dilation	DJ + nephrostomy closed	—	—

## Data Availability

No new data were created or analyzed in this study. Data sharing is not applicable to this article.
